# Attachment and School Completion: Understanding Young People Who Have Dropped Out of High School and Important Factors in Their Re-Enrollment

**DOI:** 10.3390/ijerph19073938

**Published:** 2022-03-25

**Authors:** Gro Hilde Ramsdal, Rolf Wynn

**Affiliations:** 1 Department of Social Education, Faculty of Health Sciences, UiT The Arctic University of Norway, 9404 Harstad, Norway; gro.h.ramsdal@uit.no; 2 Department of Clinical Medicine, Faculty of Health Sciences, UiT The Arctic University of Norway, 9038 Tromsø, Norway

**Keywords:** attachment theory, school dropout, school re-enrollment, social support, narrative review

## Abstract

When students drop out of high school, this is often negative for their development as well as for society, as those who drop out have an increased risk of unemployment, health problems, and social problems. The aim of the present study was to synthesize knowledge regarding processes related to school dropout in general and school re-enrollment in particular. We performed a narrative review of the literature, focusing on Norwegian and Nordic studies, but we also included studies from other countries when relevant. We discussed the findings in relation to attachment theory and our own research on the topic. As a result, we identified five main challenges to upholding education-related goals in long-term dropout processes: lack of relatedness, overchallenged self-regulation capacity, compensating for a history of failure, wounded learner identities, and coping with prolonged stress. In conclusion, the identified challenges converged on the importance of belonging and social support. The prerequisite for addressing the challenges seemed to be the establishment of a trustful relationship between the students who have dropped out and at least one teacher, and preferably also with other supportive adults. These relationships may provide sufficient social support and aid the students’ motivation to complete school.

## 1. Introduction

Formal qualifications have become a necessity for permanent employment and participation in present-day society [1]. Thus, much effort has gone into easing adolescents’ transition from secondary education into employment or higher education [2]. Most adolescents in industrialized countries complete their secondary education. In Norway, 79.6% of high school students complete high school within a 5/6 year period [3]; however, if the remaining students do not re-enroll and complete high school, this represents a huge loss for those not completing as well as for society. In Norway, the newest calculations show a financial cost of USD 1.7 million for each dropout student [4]. The students that leave school early must endure an increased risk of unemployment, incarceration, drug addiction, and becoming social security recipients [4,5,6].

The rather stable dropout rates during the last 30 years seem to challenge a basic idea of the Norwegian welfare state, namely the belief in equalizing social inequalities through education [7]. The situation in Norway reflects that of other comparable OECD (Organization for Economic Co-operation and Development) countries, where a significant minority of secondary school students are left behind [8]. The report clearly shows that young people from disadvantaged backgrounds are overrepresented in the dropout rates [8]. A systematic review could not determine which interventions were the most effective in decreasing dropout rates [9]. Interestingly, one pervasive characteristic of effective measures did stand out; at a minimum, effective interventions must establish trust and build caring relationships with the students, regardless of the context [9].

This unsuccessful search for a single effective intervention to increase school completion is most likely explained by the many factors found to influence graduation and dropout rates, including individual characteristics of students and factors associated with their families, schools, and communities [5]. Reviewing 203 studies, Lim and Rumberger [5] conclude that no single factor can explain the decision to stay in or leave school. Several salient factors within the various domains were associated with the risk of dropping out of school. Most studies were unable to establish a strong causal connection, but they did suggest a connection. The review concludes that academic achievement is one of the most widely studied predictors of school dropout, based on the indicators of test scores and grades. Grades turn out to be the most robust and consistent of the two indicators, as they subsume both ability and effort [5].

A meta-analytic review including 75 studies and 635 risk factors for school dropout found that grade retention, low IQ, learning difficulties, and low academic achievement have large effects [10]; however, the risk domains with the largest effects for school absenteeism included negative attitudes towards school, substance abuse, internalizing and externalizing problems, and low parent involvement, thus confirming that many factors are involved in school dropout processes. Moreover, various symptom profiles of psychological ill-being are also found to be associated with school dropout intentions [11].

Nevertheless, most dropout students tend to score within the normal range on IQ, despite their lower levels of academic achievement [12,13], indicating that high school graduation may not exclusively depend on inherent cognitive ability. Staying in school may also depend on how children become motivated to appreciate and master educational challenges through experiences in the parent–child relationship. In a remarkably thorough follow-up study of risk and adaption from birth to adulthood, Sroufe and colleagues [14] found that at the age of three and a half years, school dropout could be predicted with 77% accuracy from the early “quality-of-care variable”. This variable included parental responsivity to the child, parent–child attachment quality, and quality of the early home environment. In their largely lower socioeconomic status (SES) sample, neither IQ nor achievement data improved on this prediction. Sroufe and colleagues [14] conclude that it is not primarily their inherent lack of ability that is causing dropouts to leave school early. Dropouts gradually become unable to master the educational demands of school, much due to the influence of psychosocial factors, or as Finn expressed it, some children may “arrive at school predisposed to nonparticipation and non-identification” [15] (p. 130).

The importance of relationships also emerges in interview studies on school dropout processes [16,17]. When young people who had dropped out of school and stayed out of education and employment and training for 2–5 years were asked about their experiences with leaving school early, they described lives characterized by abandonment and lack of social support. They had been separated from one or both parents over longer periods of time, many had struggled to find friends in school, they could not remember one single teacher who had been supportive or helped them in mastering school tasks, and several had struggled with mental health issues after being bullied. Other dropouts described themselves as invisible at home and at school [18]. Furthermore, parenting practices are found to be related to the risk of school dropout, and even after controlling for previous academic achievement, adolescents from authoritative families were less likely to drop out than adolescents from authoritarian and neglectful families [19]. Moreover, many dropouts have been found to live in families where communication and supervision were minimal [20].

In addition, other types of relationships also seem to matter in school dropout processes. For example, an important factor in helping adolescents with mental health problems graduate from high school seems to be the teacher–child relationship. Positive teacher–child relationships were found to have the potential to reduce the association between early mental health problems and school dropout [21]. Although teacher support seems to have a particularly positive effect on school engagement and completion, relationships in general seem to play an important role in the motivational processes leading to school dropout. Both teacher support and loneliness in the school context were found to be strong predictors of students’ intention to leave school early [22].

That said, school dropout may be only a postponement of high school completion. Barrat and Berliner [23] found that about 19% of the 2011 graduating cohort had dropped out, and of these students, 22% had re-enrolled within a year; however, only 30% of the re-enrollees had graduated six years later. Other studies also indicate that early school leavers are less likely to re-enroll and complete formal learning [24,25,26]. Thus, many of the results described above indicate the relevance of early intervention in school dropout processes; however, early interventions do not always solve the problem, and some children develop problems later in their development, or their problems are discovered much later in their developmental process. These young people are also in need of some kind of intervention, some as late as after dropping out of high school. Consequently, to understand the core characteristics of a successful dropout prevention strategy, it seems essential to focus more on understanding the re-enrollment process [16,27,28].

In a prior study, we interviewed young people who had dropped out of school and work and had joined an intervention program aimed at re-enrolling them in school [29]. The participants were interviewed at the beginning of the program and again when they were about to complete it. The program offered assistance to school dropouts aged 18 to 25 in their struggle to re-enroll in and eventually graduate from high school. This was a training and support program providing long-term (more than one year) dropout students with structure and support. The participants met experienced social workers every weekday at nine for breakfast and then participated in group activities such as basketball, courses in personal economy, CV writing, and they visited potential employers, thus getting to know the local job market. The participants were provided with daily support from personnel experienced in working with young people and whose focus was on helping the participants discover their skills and find their motivation and thus gradually help them re-enroll in work or education [29].

We found that the participants who had dropped out of school and stayed out of education and regular work for long periods reported three main challenges at the beginning of their re-enrollment processes: confusion about what to do with their lives, lack of any kind of inner motivation, and lack of endurance when facing adversity. After nine months, the participants described how the intervention had strongly stimulated their inner motivation and substantially reduced their confusion about what to do with their lives. Several pointed to a re-socialization process that drew them away from watching TV and playing video games all day. The one thing they were still struggling with was resilience in the face of adversity. When problems occurred or they struggled to learn something, their first response was to withdraw into absenteeism [29]. In the aftermath of our study, we were inspired to search for relevant literature that could help us to understand these and other results on re-enrollment more thoroughly and thus contribute to the discussion on important issues in school re-enrollment processes.

## 2. Materials and Methods

There is a need for more synthesized knowledge regarding processes related to school completion. In the present study, we perform a narrative review of literature pertaining to high school dropout and re-enrollment. The purpose of a narrative review is to synthesize the literature in a particular field of study [30,31]. This is achieved by interpreting prior results narratively, pinpointing current topics and recent developments [32]. A narrative review approach is especially useful when attempting to gain an overview of a research field characterized by a high number of studies with different approaches and topics, as is the case of the dropout/re-enrollment literature. We are particularly interested in literature that sheds light on how the theory of attachment relates to high school dropout and re-enrollment.

We searched databases, including Google Scholar and PsycInfo, for literature related to our field of interest. We used different search terms, including various combinations of ‘reenrolment’, ‘reengagement’, ‘re-entry’, ‘school’, ‘youth’, ‘adolescent’, ‘child’, etc. Initial searches gave a high number of publications, and we gradually narrowed and refined our search to studies focusing on factors relating to the re-enrollment process and particularly to studies inspired by attachment theory. While we considered relevant studies from all countries, we strived especially to include relevant literature from Norway and the other Nordic countries.

The purpose of the present review is to identify and sum up central findings in the literature and to discuss these findings considering our own research relating to school dropout and school completion.

## 3. Results

### 3.1. Attachment Theory and School Completion

We have been interviewing long-term high school dropouts for the last ten years and all participants in these studies had one thing in common, namely some kind of abandonment experiences in the form of being separated from one or both parents during childhood thus lacking stable adults and social support in their lives [16,17,29]. Dropout research in general indicates that various predictors of school dropout may combine into individual disengagement processes [27]. Nevertheless, some children face different kinds of adversity and still manage to adapt and complete school [33]. The most robust predictor of such resilient adaption is supportive and responsive parenting [34]. Thus, Masten and Coatsworth [33] concluded their review of resilience research by commenting that the development of competence is protected by powerful systems, and that the quality of parent–child relationships is among the most prominent of these.

The influence of parent–child relationships on school disengagement processes has been studied from various angles: parenting practices, parenting styles, or parent–child attachment quality [35]. The most robust predictor of long-term resilience, however, is early family relationships [34]. Bowlby’s attachment theory is the most dominant approach to understanding such early family relations. Bowlby assumes that the child’s early experience of security constitutes a causal mechanism in development [36,37]. Only by increasing and upholding the proximity to the attachment figure, can the child keep safe and thereby facilitate the acquisition of skills necessary to survive [36,37]. Caretakers can either permit, ignore, or reject the child’s attachment behaviors. Based on these early interactive experiences, children develop different kinds of trust in their attachment figure. The various kinds of trust involve different expectations of support and comfort depending on the availability of the caretaker. These experiences constitute the basis for attachment quality.

Ainsworth and her colleagues explored these individual differences in attachment quality, categorizing them into secure, insecure ambivalent, or insecure-avoidant attachment [38,39]. These three different attachment qualities also define differences in children’s strategies for solving adaptational problems. Insecure avoidant children will not use the attachment figure as a safe haven, or a base for exploration, and do not seek to be comforted by the caregiver. Insecure ambivalent children will try to use the attachment figure as a safe haven by clinging to him or her, but without succeeding in establishing trust and security. Nevertheless, they all have a strategy for dealing with separation and insecurity. Some children demonstrated a lack of such a strategy through disorganized behaviors in the face of separation and were classified as disorganized or disoriented attachment [40]. Disorganized attachment was associated with high-risk environments and behavioral problems [41,42]. While Bowlby focused on the socio-emotional outcomes of variations in attachment quality, it is possible that developing insecure attachment strategies could involve a potential for disturbing the child’s basic learning processes [43].

Having no strategy or an insecure strategy for dealing with separation and insecurity, will expose these children to additional challenges in solving adaptational problems. Being less able to use their attachment figures as a safe haven, these children have less access to support in regulating their emotions. Furthermore, when children focus their attention on emotion regulation activities, they will be distracted from their exploration of the environment [44]. In the long run, such patterns may negatively influence school learning processes and academic performance, setting the children up for school disengagement and school dropout. As an example of this kind of potential mediation, Mancinelli and colleagues [45] found that maternal attachment directly and indirectly through self-control influenced adjustment difficulties. Moreover, individual differences in self-control reliably predict academic attainment and course grades [46].

Looking at parent–child attachment patterns as a causal factor in development, calls for potentially mediating mechanisms explaining the association between attachment on one side with school achievement and dropout on the other. Ijzendoorn and colleagues [43] introduced a framework of four main hypotheses for such mediating mechanisms. The attachment explorations hypothesis argues that being able to effectively reduce emotional stress, as in a secure attachment, increases the time and motivation available to explore the environment, learn to solve new tasks, and overcome problems. The attachment-teaching hypothesis states that mothers of securely attached children provide more supportive frames for child exploration and give better assistance in problem-solving situations. The social network hypothesis asserts that as early relational experiences are organized into mental representations that serve as a guide for understanding and coping with new relationships, these secure inner working models may help the child develop positive new relationships. The attachment-cooperation hypothesis declares that because secure children develop positive working models of the self and the self with others, they are consequently less anxious when away from their parents and comply better with the demands of the situation, for example, at school and in test situations. Reviewing the research related to these hypotheses, we constructed a model illustrating how many mediating influences seem to form a dynamic system of interaction [28]. The model suggests a possible mediation through all four mechanisms described by IJzendoorn and colleagues [43]. The research suggested that school dropout is a long-term developmental process influenced by early psychosocial factors that set the stage for disengagement processes.

### 3.2. Developing Education-Related Goals

One of the first things we noticed about the participants’ description of their dropout and re-enrollment processes, was their problems with developing appropriate personal goals in general and education-related goals specifically [29]; however, almost no studies have attempted to examine individuals’ personal goals over a longer period [47]. According to Salmela-Aro [47], future research should aim to create an intervention program helping in the construction of functional personal goals and building strategies for goal attainment during critical life transitions. Such construction of education-related goals was the main objective of the re-enrollment intervention program described above [29]. Although this study only followed dropout students during the nine months that the intervention lasted, the participants reported spontaneously on goal development during critical life transitions.

The participants reported a long-lasting experience of confusion about their education-related preferences [29]. They had negative experiences with school, including several unfulfilled education-related goals. Gradually, they had become ambivalent about formulating any kind of education-related goals, not wanting to disappoint themselves or others, again. Little, Salmela-Aro, and Phillips [48] declare that it is the demands, challenges, and opportunities that people encounter at a particular period in their life span that influence the kind of personal goals they construct. People seem to make choices based on these personal goals, and these goals come to influence how people manage their development. Thus, when young people become confused about their education-related goals, this might influence their ability to make choices and manage their developmental transition into young adulthood, according to a life-span model of motivation [49,50]. It is this construction of goals that will optimize or reduce a person’s potential to deal successfully with developmental transitions such as completing education and entering employment [47].

The confusion caused by faltering educational goals may also negatively influence their endurance when facing educational challenges or failures [29]. Subsequently, low academic expectations, confusion about education-related goals, and problems with endurance and adjustment of these goals in the face of adversity, as described by dropout students [16,17,29] seemed to result in what Salmela-Aro [47] (p. 66) calls a lack of “predictable, socially recognized roadmaps for human lives”. In line with this kind of thinking, intervention programs aiming at re-enrollment in high school must focus on the re-construction of these road maps.

According to the life-span model of motivation, people’s socialization can be described by the four Cs: Channeling, Choice, Co-agency, and Compensation [51,52]. When young people grow up in different environments, these various experiences will channel their developmental trajectories. Salmela-Aro [47] describes these environments as “opportunity spaces”, influencing people’s motivation, thinking, and behavior. Moreover, people are also active in their own development by making choices that influence the development of these opportunity spaces [53]. Personal goals are one factor involved in this mechanism [47]. Although young people make their own choices based on their own goals, other people are present in their opportunity space, giving them feedback and voicing role expectations and herby influencing goal construction.

The construction of personal goals is thus part of co-agency processes requiring compensations for failures and adjusting goals according to feedback. Eccles [54] suggested that these co-agency processes play a particularly important role in the construction of education-related goals and trajectories. For example, choosing to bond with friends characterized by antisocial behavior, may limit their opportunity spaces and eventually have a negative effect on academic attitudes, motivation, and school completion [55,56,57]. Accordingly, students with externalizing problems who are rejected by their teachers and their prosocial peers, seem particularly at risk of school dropout [55,58,59]. Parent–child attachment is a vital factor in the development of these co-agency processes and secure attachment has been found to promote active involvement in building effective relationships with peers and friends [60].

The young people in our studies of high school dropouts described opportunity spaces characterized by a lack of personal, parental, and teacher expectations, an excessive number of potential educational choices combined with a lack of social support [16,17]. These co-agency experiences evidently channeled their confusion about education-related goals and problems with goal adjustment, thus reducing their opportunity spaces and their choices until finally, they experienced no other option than dropping out of school. According to Wrosch and Freund [61], managing non-normative developmental demands such as school dropout requires more self-regulatory skills than managing normative events. Accordingly, Salmela-Aro [47] suggests that a lack of success with educational-related goals may act as a signal for activating goal disengagement and self-protection. Such increased demands on self-regulation followed by goal-disengagement and self-protection constitute relevant explanations to the confusion described by the participants in an intervention program aimed at high school re-enrollment [29].

The participants reported that upholding education-related goals became impossible in the face of high absenteeism and school dropout and that at the same time parents, peers, and society in general communicated that fulfilling these goals was essential. The participants described their self-regulatory skills as overchallenged by having to cope with repeated failures resulting in confusion about preferences and realistic opportunities. In addition, their self-regulation capacity was overchallenged due to their lack of experience with dividing long-term goals such as school completion into short-term goals such as completing a semester, reducing absenteeism to a minimum next month, and so on. Setting up their goal two or three years ahead meant this goal did not produce any immediate rewards. They experienced that it was impossible to activate their inner motivation over such long periods of time, without any kind of milestones to look forward to and no strategy for self-rewards; therefore, future intervention programs aimed at re-enrollment in high school should focus more on addressing the re-establishment and upholding of the participants’ trust in and strategies for realizing relevant, realistic education-related goals.

### 3.3. Intrinsic Motivation and Flow

The life-span model of motivation suggests that being unable to compensate for failures and adjust your goals, is likely to lead to depression [47]. Such compensation failures might help to explain the absence of intrinsic motivation found in our study [29]. Intrinsic motivation is supposed to bring a state of consciousness that is so enjoyable as to be autotelic (i.e., having its goal within itself) [62]. According to self-determination theory, intrinsic motivation leads to engagement [63], and the self-system model suggests that engagement is the main mediator between intrinsic motivation and academic performance [64]. A particular kind of intrinsic motivation is called ‘flow’ and is characterized by an intense and focused concentration on the present moment, merging of action and awareness, loss of reflective self-consciousness, a sense of control of one’s actions, and distortion of temporal experience [65]. The concept of flow has caught the interest of practitioners focused on the fostering of positive experiences, such as teachers in formal schooling, according to Nakamura and Csikszentmihalyi [65].

Fostering intense positive experiences is also imperative when trying to re-enroll disillusioned young people in high school and make them endure educational and social challenges. Strong positive experiences are essential to compensate for their history of failure and low self-esteem [66,67]. However, keeping up a feeling of flow is demanding due to the fragile balance between keeping the challenge interesting and rewarding without exceeding the person’s skills [65]. The importance of this fragile balance became evident, in the re-enrollment project, when staff tried to rekindle the inner motivation and education-related goals of the participants. The participants described the importance of the staff monitoring the fluctuations between boredom and anxiety tightly. Keeping participants in touch with their intrinsic motivation was described as essential for making them enroll in, engage in, endure, and complete the intervention program [29]. The all-time presence of staff and their constant follow up of the fragile balance between anxiety and depression on one side and boredom on the other were imperative to activate and develop enduring inner motivation and engagement.

### 3.4. Wounded Learners, the Re-Invention of Learning Identities

Hegna [68] interviewed male Norwegian high school students about their transition processes from being students in school, to becoming apprentices in real working life. The study emphasizes the importance of identity transformations in these processes. Although the young men did describe some positive experiences with specific teachers at school and moments of joy, these narratives were exceptions to the rule. Their experiences with school were characterized by disengagement, failure, dropout, and teachers treating them unfairly, ignoring them, administering undue punishments, or having no expectations whatsoever to their academic achievements and no confidence in them in general; however, none of these young men blamed their teachers, their school, or the system. The adolescents took full responsibility for their failure and explained them as personal failures [68].

Hegna [68] characterizes these stories as descriptions of wounded student identities, meaning that the students had stopped thinking about themselves as people who are interested in learning and able to learn. According to Hegna [68], constant negative feedback and feelings of failure substantially affected the way these young men looked at themselves and how they thought about their future. These observations are in line with the social-cultural perspective of learning, where learning and identity formation develop through a mutual interaction [69,70]. Hegna [68] suggests that, to accomplish successful apprenticeships for wounded learner identities, the workplace must offer the apprentices a learner identity that enables them to heal their wounded learner identities. More specifically, learner identity is about how students see themselves in general, but more particularly, about “how they interpret their participation and engagement with learning” [71] (p. 169). Thus, identity comes to be a precondition for learning.

What the students in Hegna’s study have in common with students in our studies are the stories they tell about their wounded student identities, the image of themselves as “not being a school person” as one of them expressed it [16,29]. The participants in the two latter studies and the apprentices from Hegna’s study, have another factor in common, namely their narratives about going through transitions. The apprentices describe transitions from school to working life, while the dropouts describe the transition from school to unemployment or the transition from being a dropout and subsequently becoming recruited into re-enrollment processes aimed at completing high school. Ecclestone [72] maintains that such transitions can become problematic when the learner identity from one context turns out to be incompatible with the learner identity necessary to succeed with the transition into another context.

Accordingly, transitioning back into school may depend on stressful identity work or becoming re-socialized into the school environment. In this line of thinking, the problem is not that they are lacking the capacity for learning, but that they have a wounded relationship with learning. Lange and colleagues [73] claim that wounded learners need to discharge and transform their former identities and make room for new and more appropriate identities. Consequently, it is a bit disconcerting that none of the dropouts we have interviewed across three studies ever described this kind of identity work initiated by teachers, counselors, or school leaders in high school [16,17,29]. Although several of them had been through two or three re-enrollment processes, there had been no attempts at transforming their wounded learner identities into more viable learner identities. Furthermore, they had received no support in changing their relationship with learning. Trying to learn from dropouts with repeated experiences of unsuccessful re-enrollment seems to indicate that the development of effective strategies to change re-enrollers’ relationships with learning is a topic in need of more systematic exploration.

### 3.5. The Management of Stress in the Re-Enrollment Process

Wounded learner identities are often explained as resulting from long-term negative stress reactions, although the concept of ‘stress’ is not often used within the field of dropout research [27]. Stress models have especially been implemented to explain the development of mental illnesses such as depression [74]. Some researchers indicate that this research underscores the relevance of stress for academic achievement and suggests the stress process as a new angle to look at dropout [75]. Dupéré and colleagues [75] specify that while high school dropout is not a mental illness, they see it as a withdrawal from stressful social situations associated with failure and/or humiliation. These kinds of external circumstances and experiences or stressors may challenge the adolescents’ adaptive capabilities and give rise to adjustment problems by restricting the ‘opportunity space’ of adolescents at risk of dropping out and those trying to re-enroll in school.

Some adolescents develop mental health problems due to such stress [17]. Lazarus and Folkman [76] state that it is the perception of lacking the necessary resources to cope with the situation that results in stress. This would imply that the experience of lacking the appropriate resources to meet the academic and social demands necessary to complete high school is one stressor in the lives of re-enrollment students; however, stressors can be both discrete disruptive events such as experiencing school dropout, and more long-term adversity such as poverty or parental drug abuse. The stress process model explains how various stressors are associated with the development of adjustment problems [75]. The model includes the unequal distribution of stressors and resources among high and low socioeconomic status groups, making some individuals more vulnerable to stress than others. Stress proliferation is a concept explaining how stress seems to accumulate. Lucio, Hunt, and Bornovalova [77] found a cumulative effect showing that the presence of at least two risk factors puts an individual at risk of academic failure. These results are consistent with the developmental model claiming that it is the accumulation of risk more than the nature of factors that are essential in predicting academic underachievement [33]. Nevertheless, reviewing re-enrollment interventions with NEETs (Not in Education, Employment, or Training), Mawn and colleagues [78] describe them to have a pragmatic approach, combining skills-based classroom training with on-the-job training. The interventions did not primarily target important psychological barriers to work engagement, such as enhancing confidence or reducing stress. Considering the high-stress reactivity during adolescence would nevertheless make the stress model highly relevant to re-enrollment research [79,80].

### 3.6. Belonging and Social Support

So far, we have suggested that understanding early psychosocial development, developing education-related goals and intrinsic motivation, mending wounded learning identities, and managing stress are central issues when trying to understand high school re-enrollment; however, none of these issues can be solved by these young people on their own. Education-related goals, for example, are developed through a co-agency process dependent on feedback [81]. This implies that dropouts in school re-enrollment processes are dependent on receiving specific types of feedback to develop education-related goals, thus making them dependent on the right kind of support. Such positive relationships are also essential for young people when they need to reduce stress, experience flow, and mend wounded learning identities. Consequently, participants in the re-enrollment program strongly stressed the importance of spending time with positive, accomplished, and helpful staff members [29]. They described how the support from these relationships helped them in their goal development, their re-motivation for school or work, in their mending of wounded learner identities through experiences of progress and success, and by assisting them in their stress management.

Hence, relationships emerge as an essential factor in several studies on dropout processes [28,82,83]. Young NEETs, who had dropped out of high school, for example, focused in their interviews on abandonment and lack of stable relationships at home and at school, describing how the absence of parents, teachers, and peers characterized their dropout processes [16]. Accordingly, Frostad, Pijl, and Mjaavatn [22] found that teacher support and loneliness in the school context were strong predictors of 16-year-olds’ intention to leave school and that loneliness outperformed well-known predictors of school completion, such as gender, parental education level, and academic achievement level.

The “caring teacher” is also reappearing as a core factor in several studies focusing on dropout processes [84,85]. The caring teachers are willing to help, hold on to high standards and high expectations, and refuse to give up on their students; however, students who drop out typically never mention any teacher that was helpful or supportive [14,17], or they describe wounded learning identities resulting from experiences with what they perceive as uncaring teachers who did not like them [86]. Consequently, dropouts and at-risk dropouts describe failed struggles to develop a positive identity in school, partly due to the absence of important relationships with peers, parents, and teachers or due to negative relationship experiences such as conflict with teachers or being bullied or marginalized.

One of the important aspects of positive relationships is the social support that they provide [87,88,89,90,91]. According to Thompson et al. [87], social support may have various functions. Social support may provide emotional support in the form of affirmation, understanding, and empathy. It may also provide social resources such as advice and guidance. Moreover, it may make information, service resources, and assistance more available. Finally, they point to the function that social support has in monitoring and detecting signs of negative development such as depressive symptoms, stressing that preventing harm is essential to promoting well-being. During stressful periods, social support can have a stress-buffering function. Social support is associated with the reduction in psychological stress, thus contributing to recovery and better coping [87]; however, only perceived support has these positive effects and, for relationships to be perceived as supportive, they need to be responsive, warm, and accepting and to provide security [92]. There seems to be a core idea that children’s interaction with parents, teachers, and peers generates generalized expectations about the self in relationships with others. These expectations are also referred to as a sense of belonging [93] or relatedness [94]. In our studies of long-term high school dropout and re-enrollment, young people describe experiences with abandonment and lack of relatedness and social support as the one thing they all had in common. They all seem to search for a climate of belongingness, recognition, and coping not sufficiently provided by their schools, their communities, or their families.

## 4. Discussion

The life span model of motivation implies that the goals individuals set are a function of the opportunities and challenges that are present in their social environment [47]. We have seen across several studies how young people coping with school dropout processes describe the various challenges present in their social environment as they struggle to construct, uphold, and fulfill their education-related goals [16,17,29]. The first challenge they describe is experiences of a lack of relatedness through, for example, abandonment and being separated from one or both parents for longer periods during childhood. In line with attachment theory, such separations and abandonment experiences may have influenced their psychosocial development and attachment quality development in negative ways. This hypothesis is strengthened by the fact that many of them describe a lack of relatedness before and during school years and in late adolescence and early adulthood. Using the perspective of the life span model of motivation, such challenges to their relatedness may have restricted their opportunity spaces from an early age, thus negatively influencing their construction of education-related goals.

According to the model, co-agency processes and feedback are of particular importance to the construction of education-related goals [47], indicating the importance of stable and competent adults and the presence of social support. The lack of success in constructing and upholding education-related goals, may act as a signal for the goal disengagement and self-protection processes seen in our interviews with long-term dropouts [16,29]. Managing such non-normative developmental demands as those associated with school dropout, seems to require more self-regulatory skills than managing normative school completion processes [61] and thus defining a second challenge to upholding education-related goals. According to Bowlby [37], the development of self-regulatory capacity is a consequence of early parent–child interaction and emerging attachment patterns; however, for the young people in our studies describing abandonment, lack of relatedness, and social support as characteristics of their school dropout processes, these excessive demands on their self-regulatory capacity may have overchallenged their ability to self-regulate. This may have reduced their ability to handle academic failures and the succeeding blows to their academic self-concepts and their self-esteem.

In line with this thinking, Salmela-Aro [47] suggests that such an inability to compensate for failures and thus adjust your goals is likely to lead to depression. According to Baumeister et al. [66], strong positive experiences are needed to compensate for a history of failure and low self-esteem. Fostering such intense positive experiences emerge as a third challenge when trying to remotivate and re-enroll disillusioned and disengaged young people in school. Keeping participants in touch with their intrinsic motivation was described as essential for long-term dropouts in completing a school re-enrollment program [29,95]. According to these participants, keeping them in touch with their inner motivation depended upon the establishment of trusting relationships to, and close follow-up by, competent adults. The participants also describe how they became re-socialized as they were offered new learner identities, thus enabling them to heal what Hegna [68] called their wounded learner identities, a fourth challenge to upholding or adjusting education-related goals.

Consequently, such a wounded relationship with learning seems to imply that challenging identity work is a precondition to succeed in the re-enrollment of dropouts in school. Several identity theories propose that peoples’ identities are formed according to how they perceive others perceiving them [96,97], implying the importance of relatedness in identity formation and change. Furthermore, the concept of wounded leaner identities suggests that dropout also implies a fifth challenge, the management of stress through withdrawal from stressful social situations associated with failure and humiliation. The participants in our interviews of long-term dropouts often described to us how one of the characteristics involved in their wounded learner identities, is their experience that teachers and peers and eventually the participants themselves perceive them as lacking the necessary resources to cope with the situation, and how this perception contributed to prolonged stress and failure to uphold their education-related goals [16,17,29].

This review has both strengths and limitations. We have pointed to the need to gain an overview of the literature on re-enrollment. The method of narrative review was chosen in order to evaluate a high number of studies with different topics and approaches relating to school dropout and re-enrollment and to synthesize the literature. The process of synthesis rests on the ability of the authors to search, analyze, and summarize the relevant literature. While our approach hopefully has succeeded in producing an overview and understanding of the literature on re-enrollment, there is also a risk that some relevant literature has not been included or that its importance has been underplayed as the literature within this field is considerable in its volume and diversity.

## 5. Conclusions

We set out to understand more about these high school re-enrollment processes. Focusing on relevant theories and prior research, including our own research, we have identified five main challenges to the motivational process of upholding education-related goals in long-term dropout processes. These challenges are: lack of relatedness, overchallenged self-regulation capacity, compensating for a history of failure, wounded learner identities, and coping with prolonged stress; however, all of these challenges seem to converge on the importance of belonging and social support. The prerequisite for addressing all these challenges seems to be the establishment of a trustful and warm relationship between the students who have dropped out and at least one teacher, and preferably also with other competent and supportive adults who are present in their everyday lives for a substantial period of time. These relationships may provide sufficient social support to aid the students’ inner motivation.

## Data Availability

Not applicable.
